# Translating and applying a simulation model to enhance understanding of grassland management

**DOI:** 10.1111/gfs.12584

**Published:** 2022-09-27

**Authors:** Michail L. Giannitsopoulos, Paul J. Burgess, Matthew J. Bell, Goetz M. Richter, Cairistiona F. E. Topp, Julie Ingram, Taro Takahashi

**Affiliations:** ^1^ School of Water, Energy and Environment Cranfield University Cranfield Bedfordshire UK; ^2^ Department of Agriculture Hartpury University HEC Gloucester Gloustershire UK; ^3^ Rothamsted Research Sustainable Soils and Crops Harpenden Hertfordshire UK; ^4^ Scotland's Rural College Peter Wilson Building, King's Building Edinburgh UK; ^5^ Countryside & Community Research Institute University of Gloucestershire Gloucestershire UK; ^6^ Rothamsted Research, Net Zero and Resilient Farming North Wyke Okehampton UK

**Keywords:** agronomy, decision support, education, grassland management, LINGRA

## Abstract

Each new generation of grassland managers could benefit from an improved understanding of how modification of nitrogen application and harvest dates in response to different weather and soil conditions will affect grass yields and quality. The purpose of this study was to develop a freely available grass yield simulation model, validated for England and Wales, and to examine its strengths and weaknesses as a teaching tool for improving grass management. The model, called LINGRA‐N‐Plus, was implemented in a Microsoft Excel spreadsheet and iteratively evaluated by students and practitioners (farmers, consultants, and researchers) in a series of workshops across the UK over 2 years. The iterative feedback led to the addition of new algorithms, an improved user interface, and the development of a teaching guide. The students and practitioners identified the ease of use and the capacity to understand, visualize and evaluate how decisions, such as variation of cutting intervals, affect grass yields as strengths of the model. We propose that an effective teaching tool must achieve an appropriate balance between being sufficiently detailed to demonstrate the major relationships (e.g., the effect of nitrogen on grass yields) whilst not becoming so complex that the relationships become incomprehensible. We observed that improving the user‐interface allowed us to extend the scope of the model without reducing the level of comprehension. The students appeared to be interested in the explanatory nature of the model whilst the practitioners were more interested in the application of a validated model to enhance their decision making.

## 1 INTRODUCTION

In the UK, because about 75% of ruminants' dry matter consumption originates from either grazing pasture, grass silage or hay (Wilkinson, 2011), successful grassland management is an important determinant of the profitability of dairy, beef and sheep production. Successful grassland management, in turn, hinges on a farm manager's ability to modify fertilization, grazing and harvesting practice to optimize the yield and quality of grass forage in response to the effects of weather, soil type, and soil nutrient status. However, enabling a deep understanding of these relationships, particularly for each new generation of farmers, is a challenge. As these multiple interactions can neither be completely observed nor their consequences be accurately foreseen (Takahashi et al., 2018), computer simulation models can provide useful tools to demonstrate how grass production responds to different environment conditions and management (Movedi et al., 2019; Pulina et al., 2018; Qi et al., 2018).

### 1.1 Digitization and the role of computer models in agriculture

Computer models have been widely used in research, relating crop growth to management and the environment, developing systems‐level thinking, and exposing knowledge gaps (Boote et al., 2001; Goudriaan, 1996; Hammer et al., 2002). Recent years have also seen the increased use of digital technology and models to guide decision making for grassland at field, farm, and policy levels. Projects such as GrassCheckGB (https://grasscheckgb.co.uk/) are helping farmers to benchmark and forecast grass resources on farm, for instance, whether to allocate particular fields for grazing or silage production (Barrett et al., 2005; Korhonen et al., 2018; Ruelle et al., 2018). There is an established literature on the usefulness of Decision Support Tools derived from models for land managers (Keating & McCown, 2001). Models can help policy makers identify if changing weather conditions necessitate the need for farm income support and to determine long‐term effects of climate change, which cannot be answered through field experiments (Graux et al., 2013; Kipling et al., 2016a; Mobbs et al., 2001). A third potential use of crop models is as tools for teaching and prompting learning, and this has generally received less attention.

### 1.2 Use of models to support teaching and prompt learning

As a teaching tool, it has long been recognized that computers and crop simulation models can provide training opportunities for undergraduates to improve their understanding of crop development and growth processes (O'Shea & Seld 1983; van Ittersum et al., 2003), particularly where the teacher has confidence in the model (Jamieson‐Proctor et al., 2013).

As a tool to prompt learning, models can support in two ways. In the field through decision support, they can complement practitioners' own adaptive management process whereby they adjust their decisions and actions based on experience and feedback (Norton & Reckhow, 2006). Here, computer models can be used to support short‐term immediate choices or inform long‐term strategic plans. They can also be used in participatory settings to prompt social exchange. For example, grass‐based simulation games have been used to stimulate discussion and reflection on how grassland management in France may alter with climate change (Martin et al., 2011).

The success of a tool for teaching or prompting learning for students or practitioners respectively has been related to a range of attributes. These can be distinguished for the different user‐contexts. In an education context, tool flexibility is useful (Thomas & Neilson, 1995) and model transparency can allow students to examine both the structure and the processes of the model (Sinclair & Seligman, 1996). For practitioners, important attributes again include flexibility to fit the complexity of farm environments and decision making, minimal data requirements, and credible and meaningful outputs which reflect users' experiences (Hayman & Easdown, 2002; Matthews et al., 2008; Smith et al., 1997). Although associated with decision making, these attributes are closely interrelated to learning (Lundström & Lindblom, 2018). The ability of tools to prompt discussions and allow the user to ask ‘What if?’ questions is regarded as a fundamental characteristic of learning in tool use (McCown et al., 2012).

The context for this study was that we observed that there was no freely available grassland‐growth model in the UK that could be used as a tool for teaching or learning to examine the effect of grassland management on grass yields. Although computer‐based grass growth simulation models have been used in research (Barrett et al., 2005; Qi et al., 2017), these had not been translated into use in the classroom or with practitioners in demonstration and discussion workshops. Hence the aim of this paper is to describe the process of developing a grassland simulation model, specifically the testing, evaluating and refining of it in student and practitioner workshops.

## 2 METHODOLOGY

**FIGURE 1:**
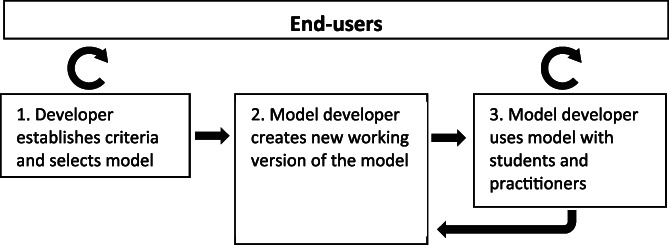
Schematic illustration of the (1) selection, (2) development and (3) evaluation and iterative improvement of the model

The methodology comprised a process of (1) selecting a suitable model framework, (2) translating and developing the model and then working with potential users in a series of workshops in an iterative process to evaluate its potential for teaching and learning and to refine it based on feedback (Figure 1). The most recent version of the model, called LINGRA‐N‐Plus, is freely available on‐line (Giannitsopoulos et al., 2020), together with a teaching guide. A full description of the technical aspects of the model and its validation with measured data is provided by Giannitsopoulos et al. (2021).

### 2.1 Selection of model

There is a wide range of grass growth simulation models that have been developed for research. However, for our purposes, the two principal criteria for selecting a grass model as tool for teaching and learning were (i) the availability of supporting documentation and (ii) the capacity to translate the model into a Microsoft Excel spreadsheet interface. Translation to a spreadsheet was considered desirable as the software environment is widely used by students and it is relatively easy for students and practitioner users to create graphs and tables for the associated outputs (Niglas, 2007).

Based on the above criteria, we selected the LINGRA model (LINtul GRAssland; Schapendonk et al., 1998), which has been developed in the Netherlands and subsequently used to predict growth and development of perennial ryegrass across the European Union (Joint Research Centre, 2018) for both potential and water‐limited growing conditions. The model was originally programmed in Fortran (Wolf, 2006), but in 2014, Aart van der Linden re‐wrote LINGRA in the widely used ‘R’ programming language. In the UK, variants of the LINGRA model have been used to describe the growth of other grass species such as switchgrass (*Panicum virgatum*) or miscanthus (*Miscanthus* giganteus) (Ni et al., 2019; Triana et al., 2011) and used in upscaling studies to determine the effect of different grassland types and management intensities on UK grass production (Qi et al., 2017, 2018). LINGRA has also been modified to address grass survival during the winter at high latitudes (Höglind et al., 2016) and the specific characteristics of the grass species timothy (*Phleum pratense L*.) (Höglind et al., 2001; van Oijen et al., 2005).

### 2.2 Initial development of a tool for teaching and learning

**FIGURE 2:**
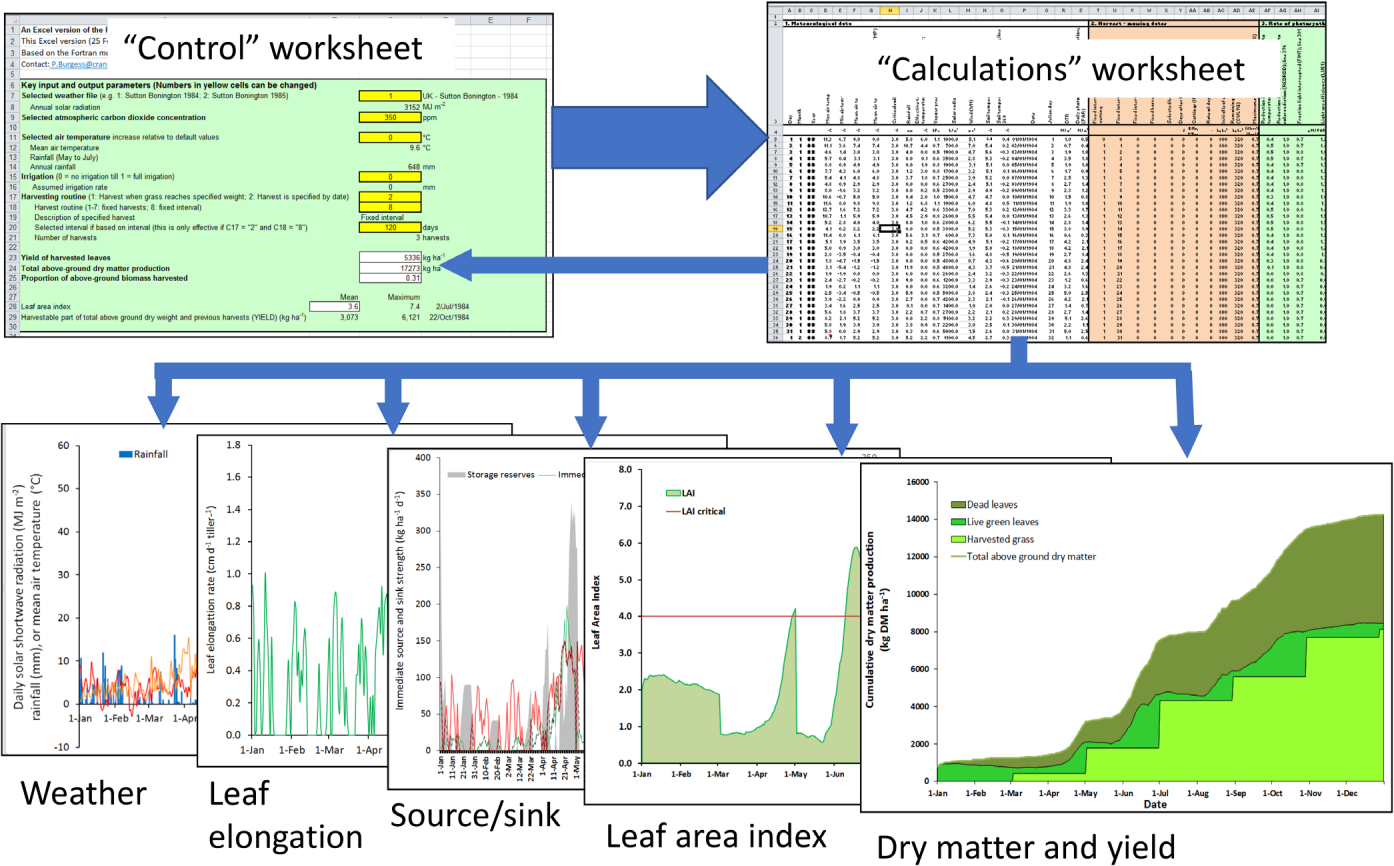
Schematic illustration of LINGRA learning tool: Key worksheets include the ‘control’ the ‘calculations’ and 13 worksheets displaying graphical representations of the outputs

Because of the excellent documentation associated with the ‘R’ version of LINGRA (Wolf, 2006), it was possible to create a spreadsheet‐version of the model in Microsoft Excel, familiar to students and some practitioners, that allowed the easy creation of graphical outputs. The developed LINGRA model in Excel included one ‘Control’ worksheet, one ‘Calculations’ worksheet, and 13 worksheets displaying graphical representations of the outputs (Figure 2). There was also a sheet that stored a range of ‘weather’ inputs and a sheet describing carbon dioxide concentrations for different years. The ‘Control’ worksheet was designed so that the user can select pre‐determined default options including specific sites in specific years (defined in terms of daily weather data), a choice of atmospheric CO_2_ concentrations and air temperatures relative to default values, and management options such as harvest dates (Figure 2). The ‘Calculations’ sheet, which is the engine of the model, uses a daily timestep and each day in the year (1 January to 31 December) appears as a separate row. The same algorithms are used for each day. The ‘Calculations’ worksheet uses (1) the weather data and data in the ‘Control’ worksheet to calculate (2) harvest dates, (3) rates of photosynthesis, (4) the water balance, (5) leaf appearance and extension rates, (6) leaf death rates, (7) dry matter production and reserves, and (8) evapotranspiration rates.

The choice of cutting dates can be specified in the ‘Control’ worksheet either as specified dates or it can be based on a predetermined crop weight (e.g., the grass is cut when it reaches a weight of, for example, 1500 DM kg  ha^−1^). Alternatively, the user can also opt for cuttings at specific time intervals, e.g., every 20, 40, or 60 days between a specified start and end date. Another variable is the level of irrigation; the user can indicate whether the crop is irrigated (1 = irrigated) or grown as a rainfed crop (0 = no irrigation).

### 2.3 Evaluation of the LINGRA model and incorporating feedback

Evaluation was undertaken in three phases over a period of 12–18 months. Two groups were selected to determine if they had different experiences with and perspectives of the model: (1) students (as learners and as potential future grassland managers) on undergraduate agricultural courses and (2) practitioners‐ current grassland farmers, consultants, and advisors (Table 1).

**TABLE 1:** Initial and second set of workshops to use and evaluate the grass model

Workshops	Participants	Workshop location	Date	Attendees	Respondents
Initial model	Students	Nottingham, England	February 2019	30	14
	Practitioners	Dumfries, Scotland	March 2019	11	8
		St. Asaph, Wales	October 2019	4	4
		Edinburgh, Scotland	November 2019	3	2
Updated model	Students	Nottingham, England	February 2020	30	17
	Practitioners	Cranfield, England	February 2020	12	7
		North Wyke, England	February 2020	6	6
		Nottingham, England	February 2020	12	12
Total				108	70

Phase 1: The initial version of the LINGRA model in Excel was used with and evaluated by these two groups in 2019: Workshops were held with students at the University of Nottingham, practitioners (Scotland's Rural College (SRUC) dairy consultants at the SRUC campus in Dumfries) and grassland consultants and farmers (FarmConnect and independent) at St Asaph in North Wales.

Phase 2: Feedback was incorporated and the tool was revised by the research team to create a new version (LINGRA‐N‐Plus). A workshop was held with a group of researchers at the SRUC campus in Edinburgh, Scotland to validate this (Table 1).

Phase 3: The revised version of the tool (LINGRA‐N‐Plus) was used with and evaluated in four workshops in February 2020. One workshop with undergraduate students at University of Nottingham who were completing a grassland management module, one with researchers at Cranfield University, one with grassland consultants and researchers at North Wyke in Devon, one with grassland advisors and researchers convened at Nottingham University. The total number of participants was 108 (Table 1).

**FIGURE 3:**
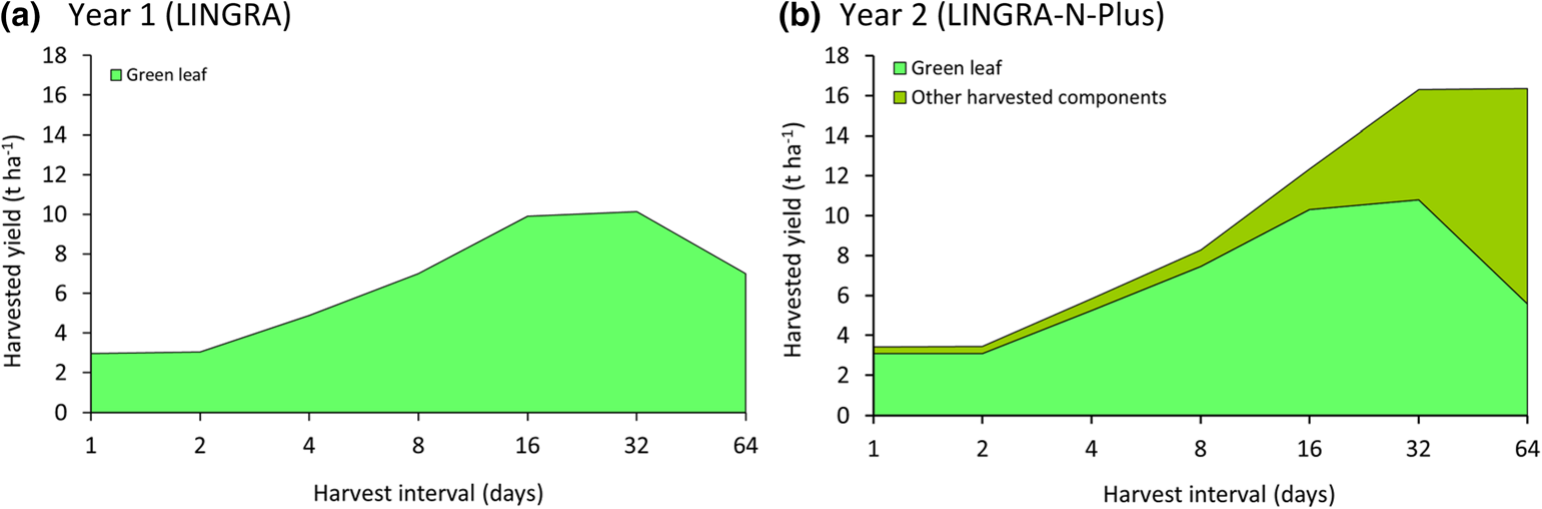
Modelled effect of harvest intervals on (a) the harvestable yield of green leaves in year 1, and (b) both the harvestable yield of green leaves and other components (stems, dead leaves and seeds) in year 2, using weather data from Sutton Bonington for 1985. Note that the *x*‐axis is not linear

The 2 h workshops in Phases 1 and 3 followed the same format. The workshop started with a model introduction and demonstration supported with a worksheet/guide. The model was initially used to describe the effect of different harvest intervals on the yield of the green leaves. This use highlighted that there was an optimal harvest interval to maximize the yield of green leaf: too frequent and grass growth was unable to fully recover between harvests; not frequent enough and the green leaves would die before harvest (Figure 3a). Other uses explored with participants included the effects of different weather patterns, carbon dioxide concentrations, irrigation or drought stress, and different soil depths. Following this, the participants were encouraged to use, explore, and evaluate the model in a ‘hands‐on’ way using annual weather data from a local site and inputting their own or hypothetical management practices (what if).

Based on relevant themes in the literature, a framework with four key attributes (Table 2) was developed to steer the workshop evaluation. This was used to frame questions to the participants and steer their discussions, both following the demonstration and at the end of the exploratory session. It was not possible to directly evaluate learning in terms of knowledge acquired through use of the tool, however proxies for learning were identified from the literature, also the attributes of user experience and relevance/usefulness all influence the users' potential learning and teaching.

**TABLE 2:** Framework for analysis of workshop discussions

Main theme	Criteria used to prompt workshops questions	Literature sources
User experience	Tool performance, ease of use‐interface/control sheet	Hayman & Easdown (2002); Lundström & Lindblom (2018); Mathews et al. (2008); Matthews et al. (2011); Smith et al. (1997)
	Data requirements	
	Robustness	
	Simplicity/complexity and completeness of relevant detail	
	Transparency	
Relevance/usefulness	Usefulness for different users. Meaningful outputs and format	
	Potential to support management decisions and planning	
Teaching	Improves student understanding of factors that affect grass growth	van Ittersum et al. (2003); Sinclair & Seligman (1996)
	Allows students to examine model structure and processes	
	Stimulates discussion in participatory tool development.	
Learning	Improves understanding	Martin et al. (2011); McCown et al. (2012); Lundström & Lindblom (2018)
	Prompts ‘what if’ questions	

At the end of each workshop, the participants were given an evaluation sheet. These were purposely kept as open as possible and were framed by strengths, weaknesses and opportunities comprising three questions: (1) ‘What were the strengths of the model and the session?’, (2) ‘What were the weaknesses of the model and the session?’ and (3) ‘What opportunities do you see for the next stage?’. All responses were anonymous, although participant type was noted. Overall, 70 out of a total of 108 participants provided written feedback (a response rate of 65%; Table 1).

Analysis of workshop outputs comprised (i) the analysis of qualitative data collected in the workshops: participant observation, transcripts of workshop discussions and (ii) the qualitative analysis of participant evaluation sheets. The initial LINGRA spreadsheet model was evaluated in four workshops, by one group of students and three groups of practitioners (total number of 28 respondents; Table 1).

## 3 RESULTS

### 3.1 Phase 1: Feedback on the initial LINGRA grass tool

#### 3.1.1 Workshop discussion

##### User experience

Participants appreciated the ease of use and modification of input parameters. They liked the tool's ability to demonstrate how management decisions, such as cutting intervals affected yield, in particular the graphical visualization. Both students and practitioners suggested including other variables especially soil factors such as temperature, pH and organic matter. The lack of response to *N* was considered by all as a limitation. It was also suggested to include crop characteristics and management factors as well as to incorporate weather forecasts and financial analysis. Practitioners were interested in having a simpler version or a simplified interface of the tool compared to students.

##### Relevance/usefulness

Participants broadly agreed that the tool had merits for students but saw less value for farmers and consultants who already know about management and understand the effect of management decisions. For most farmers, according to participants, a 21‐day harvest interval was considered the appropriate average for the context, and those using shorter or longer intervals generally know what they are doing and do not need the tool.

##### Teaching and learning

Students and practitioners appear to use the tool differently. Students interrogated causal processes by asking ‘how?’ and ‘why?’. For example, when examining how changes in rainfall or harvest interval affect green leaf yields in general terms, they place greater emphasis on the interactive nature of the tool. Rather than looking at the tool from the perspective of factors that change grass yield, the practitioners assessed it in terms of management decisions. In this respect they tended to seek more quantified information for specific circumstances, asking ‘how much?’ and ‘when?’. For example, what is the typical yield increase when the harvest interval is increased from 14 to 21 days for specific soil and weather conditions?

#### 3.1.2 Feedback on the evaluation sheet

The feedback expressed in the evaluation sheet confirmed the analysis of the workshop discussions. In terms of the model's strengths, participants placed emphasis on its capacity to demonstrate how management decisions affected yield (10 out of 28 participants in 2019; Table 3), the capacity to immediately visualize outputs in graphical form (8), its engaging and interactive nature (8), and the ease of using and changing input parameters (7). Four users recognized the strength of the model in the speed of calculations and the benefits of using a widely available spreadsheet platform, while three participants considered it useful for teaching or demonstration. In terms of weaknesses, 18 out of the 28 users commented that it would be beneficial if the tool integrated more variables, such as grass response to nitrogen (N) application and the effect of management on grass digestibility and crude protein. Seven participants indicated that the model was too confusing or complex and three participants wanted the session to be more interactive. One person commented on the lack of a step‐by‐step guide and two participants highlighted the benefits if the model could also be more ‘predictive’. In terms of opportunities for improvement, 14 participants highlighted that, additional variables could be useful, whilst six indicated that a simpler model version would be helpful. Four users indicated that additional input data and an evaluation of the outputs with measured grass yields might be needed. Finally, three participants supported the idea of using the model with various end‐user groups and one person highlighted opportunities to use the model within another grassland research project.

**TABLE 3:** Users' comments (receiving more than 1) of the LINGRA tool in 2019 and the LINGRA‐N‐Plus tool in 2020, and the cumulative total for students and consultants

Number of returned evaluation sheets	LINGRA tool (2019)			LINGRA‐N‐Plus tool (2020)			Students *n* = 31	Practitioners *n* = 39
	Students *n* = 14	Practitioners *n* = 14	Total *n* = 28	Students *n* = 17	Practitioners *n* = 25	Total *n* = 42		
Strengths								
Demonstrates how inputs affect yield	4	6	**10**	6	7	**13**	10	13
Visualization (excellent graphs)	2	6	**8**	1	7	**8**	3	13
Engaging, interactive, informative model/session	5	3	**8**	5	2	**7**	10	5
Easy to use/understand and input data	4	3	**7**	8	9	**17**	12	12
Shows grassland complexity		5	**5**	1	3	**4**	1	8
Speed of calculations/Excel availability	2	2	**4**		2	**2**	2	4
Excellent for teaching/demo tool		3	**3**	2	11	**13**	2	14
Input information are all available		3	**3**	2	2	**4**	2	5
Unique/enjoyable/beautiful	2		**2**	2	1	**3**	4	1
Weaknesses								
Integrate more variables	6	12	**18**	5	8	**13**	11	20
Can get confusing/too complex	2	5	**7**	3	6	**9**	5	11
In practice, weather inputs are confounded	3		**3**	1		**1**	4	
More interactive	3		**3**				3	
Locally relevant input data and validation		2	**2**					2
It is not predictive	2		**2**				2	
Intent to use with different end‐users	1		**1**		2	**2**	1	2
Provide/improve step by step guide	1		**1**	2	1	**3**	3	1
Amend/simplify control worksheet				3	12	**15**	3	12
Opportunities								
Integrate more variables	3	11	**14**	16	17	**33**	19	28
Additional input data and validations	1	3	**4**	1	3	**4**	2	6
Intent to use with different end‐users	1	2	**3**		2	**2**	1	4
Simpler version		6	**6**					6
Integrate with other systems		1	**1**		4	**4**		5
Can be more than a teaching tool				1	6	**7**	1	6
Amend/simplify control worksheet				1	5	**6**	1	5
Develop an App/website				2		**2**	2	

*Note*: The numbers in bold are simply the sum of the value for students and the value for practitioners.

### 3.2 Phase 2: Improving the model in response to initial feedback

Following the initial feedback reported above, we identified five actions to enhance the model. Two of the actions were teaching‐based and subsequent sessions were planned to involve greater ‘hands‐on’ activity and a teaching guide was produced (Burgess et al., 2020).

In response to suggestions from both students and practitioners, basic processes of *N* dynamics were included and the third action was to integrate a yield response to *N* inputs within the model. The grass response to *N* was initially included using algorithms from the LINGRA‐N model (Wolf, 2012). However, an initial evaluation showed that modifications were needed to the *N* uptake dynamics that are determined by the *N* demand of leaves and stems, the initial soil mineral *N* status, the amount of available mineralisable soil organic *N* (Addiscott & Whitmore, 1987) and the amount of *N* applied as fertilizer. The *N* recovery fraction was assumed as a fixed proportion (0.7) of applied *N*. Because of these changes, the updated model was named ‘LINGRA‐N‐Plus’ to distinguish it from the existing LINGRA‐N model.

The original LINGRA simulated only green leaves, whereas in practice grass yields also include stems and dead leaves (Wilman et al., 1976). Additional algorithms were included within the LINGRA‐N‐Plus model in the fourth action, so that the proportions of dry mass allocated to green leaf, stem, and seeds varied as a function of thermal time from the last harvest. New algorithms were also included to determine the proportion of standing stem and dead leaves that were removed at each harvest. It was assumed that the proportion of standing stems harvested would be the same as that for green leaf. Using data from Wilman et al. (1976), we also assumed that the weight of dead leaves harvested (expressed as a proportion of the total weight of green leaves and stems) would be zero below a harvest interval (HI) of 21 days, be equal to 0.0035 * (HI‐21) for intervals between 21 and 70 days, and then reach a plateau value of 0.1715 for intervals above 70 days.

**FIGURE 4:**
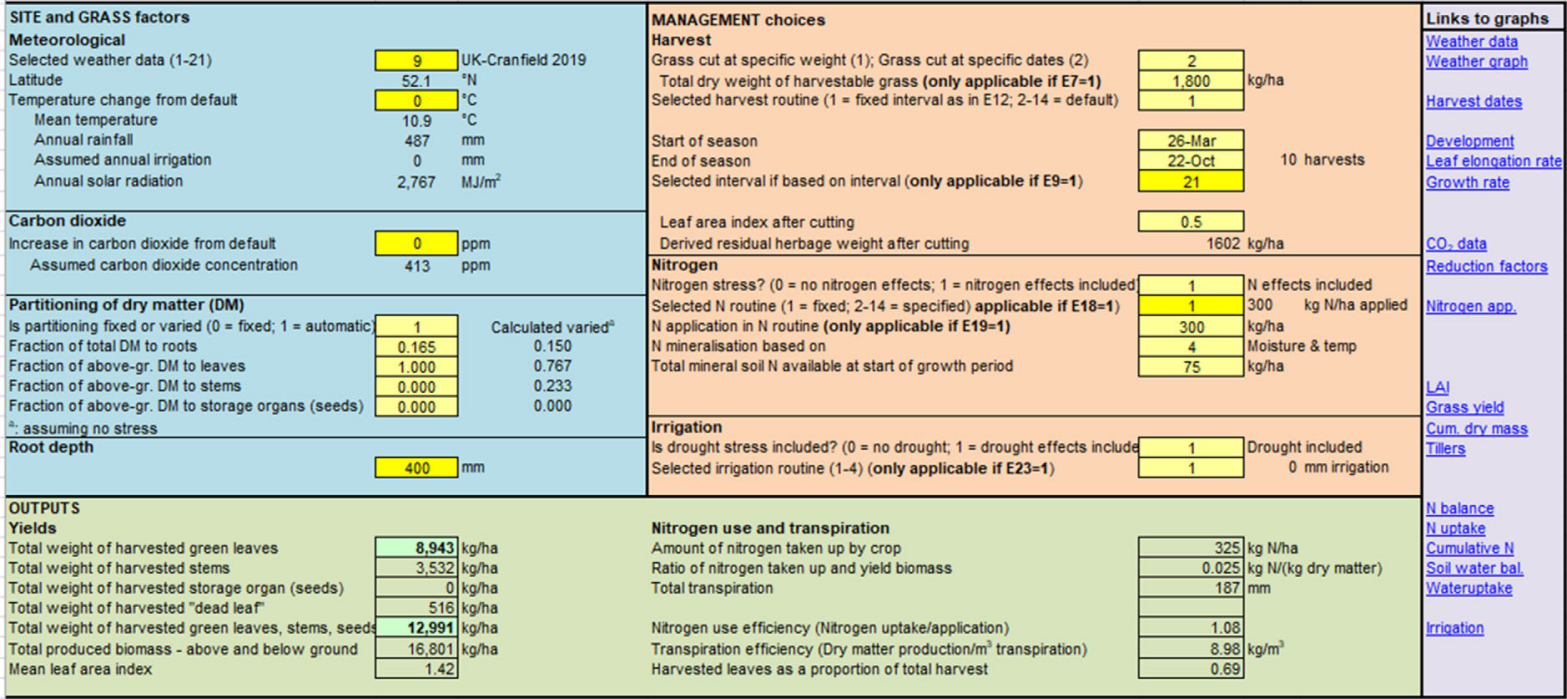
LINGRA‐N‐Plus screenshot important inputs and important outputs section of the Control worksheet as set for Cranfield 2019 weather data.

The fifth action was to improve the user‐interface by restructuring the ‘Control’ worksheet so that key inputs or outputs were clearly categorized under the headlines of ‘Site and grass factors’, ‘Management choices’ and ‘Outputs’ (Figure 4). ‘Site and grass factors’ included site choice, meteorological data, atmospheric carbon dioxide concentrations and assumptions regarding the partitioning of dry matter by herbage. The ‘Management choices’ section included the choice of different cutting intervals and cutting intensities, *N* and irrigation rates. The updated ‘Outputs’ section describes the dry matter yields of different grass parts (leaves, stems and total) and summarizes *N* use and transpiration.

### 3.3 Phase 3: Evaluating the implementation of LINGRA‐N‐Plus

#### 3.3.1 Workshop discussion

##### User experience

The overall experience was more positive than in Phase 1 in terms of ease of use and the tool's ability to demonstrate the effects of management decisions. Although improved *N* dynamics were included in the revised model, participants identified additional factors to be included such as the effects of soil pH, phosphorus and potassium levels (soil and fertilizer) and soil organic carbon dynamics. However, while more variables were identified for inclusion, participants often also call for more simplicity and described the tool as complicated. The workshops again prompted detailed conversations about cutting intervals based on the practitioners own experiences and identified other considerations such as nutritional value and the significance of the leaf to stem ratio. There was also interest in describing the grass remaining after cutting in terms of a ‘residual weight’ and relating the different leaf and stem yields to a feed value. The possibility that the model could simulate different grass species and the effect of defoliation on roots was also discussed. The consultants suggested using parameter/metrics that farmers are familiar with and can visualize e.g., total dry matter not green leaf, arguing that farmers want to harvest at a certain biomass of kg dry matter and that leaf area index (LAI), for example is not a suitable metric for them.

##### Relevance/usefulness

Some questioned the provenance of the model and argued that it was not relevant to farmers if they grow native species, herbal leys or mixed species. However, the opportunity to use it with beef and sheep farmers who do not practice rotational grazing, to change people's way of thinking by showing them the impact of cutting at set intervals was recognized. The value of the tool for strategic planning was suggested, asking long term ‘what if’ questions.

##### Teaching and learning

There was a positive response to the tool's ability to enhance understanding of grass growth processes. The value of being able to see the ‘workings’ for students was emphasized, although it was not considered as important for practitioners. However, some consultants and advisers liked the idea of using the model with various groups of farmers as a learning tool, arguing that the current tools are a ‘blackbox’ and they find it difficult to encourage discussion around them. All the conversations shared in the workshops are in themselves indicative of the tool's ability to prompt shared learning amongst participants.

#### 3.3.2 Feedback on the evaluation sheets

The analysis of the evaluation sheets confirmed the workshop discussions. From user feedback, the highest positive response (17) was that LINGRA‐N‐Plus was easy to use, to understand and input data (Table 3). As in the initial workshops, participants highlighted that the model could be an excellent teaching or demonstration tool (13), and that it could immediately demonstrate how inputs affect yield (13). Some participants described the tool as unique, enjoyable, or beautiful (3).

The participants highlighted that one of the weaknesses of the model was that it still needed to integrate more variables (13), whilst nine out of 42 respondents found the updated tool too complex or confusing. A total of 15 participants highlighted that the control worksheet could be further simplified. In terms of future opportunities, 33 participants highlighted the scope to include more variables. Seven participants indicated the potential of LINGRA‐N‐Plus as a learning tool.

## 4 DISCUSSION

The observations we made while developing and applying the tool, and analysing the workshop discussions and evaluation sheets, can be grouped into the following categories of (1) its ease of use, (2) the importance of visualization, (3) the compromises between simplicity, transparency and complexity, (4) the different perspectives of users, (5) the effectiveness of pedagogy, (6) how stakeholder involvement improves model design and is a mechanism to facilitate learning, and (7) limitations and future developments of the tool.

### 4.1 Ease of use

The model was recognized as being easy to use. The three features of the model that supported its ease of use are (i) the use of a familiar software environment, (ii) the inclusion of a well‐structured control worksheet, and (iii) the capacity to access relevant inputs. These features are discussed in turn.

The most appropriate platform can depend on availability, costs, suitability for the task, knowledge of the developers, and the ability to transfer and utilize models between users (Graves et al., 2005; Voinov & Bousquet, 2010). In this case, Microsoft Excel was an appropriate choice, particularly in the sessions with students where their familiarity with the software allowed them to quickly navigate through the main features of the model implementation. The model could be used on the participants' laptops at each event, and there were no problems with processor speeds.

However, an open spreadsheet format can have disadvantages. The open format means that it is possible for a user to enter a value or equation in the wrong place, thereby disrupting the whole model. However, this issue could be solved by using password protection in certain sheets or cells containing equations. The spreadsheet environment was also not set up to limit inputs, for example negative values, to specific cells. However, this issue could be solved by a model developer adding error checks to prevent the entry of obviously erroneous values. Although an open format has the advantage of allowing a user to develop new bespoke versions of the model, it is also important to maintain a ‘master’ version. Hence a master version of the model has been made available in an online repository (Giannitsopoulos et al., 2020).

The use of a ‘Control’ worksheet to function as a ‘dashboard’ for the rest of the model worked well. Such a Control worksheet allowed the user to see the most important input and output variables on a single screen. Jame & Cutforth (1996) also highlighted the usefulness of a dashboard to increase the usability and utility of a model. Ease of use was also supported by the use of pre‐entered input data. In both sets of workshops, the model included local weather data preloaded within one of the spreadsheets. This accessibility of input variables, including default soil and initial grass state variables, made the model relatively easy to use.

### 4.2 Importance of visualization

Accessible outputs are important for the user to understand the model results (Hamilton et al., 2019). Responses from LINGRA‐N‐Plus users highlighted the importance of visualization in learning, for example they could instantly observe the effect of varying inputs, such as changes in rainfall pattern or harvest frequency on yield. Such data visualization can provide a bridge between the quantitative content of data and human intuition (Donalek et al., 2014) making complex data easier to understand, both, for education and expert analysis (Christensen et al., 2018).

The capacity to use the model interactively allowed the user to explore, experiment, hypothesize, practice and test a range of phenomena or assumptions. Although on‐line electronic teaching resources can be used individually outside of the classroom (Stančić et al., 2007), integrating the teaching tool with farm visits, grass biomass measurements, video recordings and discussions with farmers could benefit the students.

### 4.3 To add more variables or to simplify?

Both students and practitioners identified a number of additional variables to include as well as accounting for nutritional aspects for example. Some of these processes could be implemented relatively simply, but others are more complex (Kipling et al., 2016a). How does a model developer identify the optimal number of variables within a tool intended for learning? Whilst a substantial number of students and practitioners requested the inclusion of more variables, others indicated that the tool was too complex and confusing. Chwif et al. (2000) emphasized that within the modelling community, there is often a stated preference for simple rather than complex models. For example, Pidd's (1996) second principle of modelling was to be parsimonious, start small and only to add additional details if they were needed, which is in line with Passioura (1973) and Wainwright and Mulligan (2013). Chwif et al. (2000) highlighted that the appropriate complexity of a model should be a function of the detail required in relation to the scope of its application. A model using a daily time‐step is likely to be more complicated than one with an annual time‐step. Likewise, a model describing yield and quality will be more complicated than one only describing yield.

**FIGURE 5:**
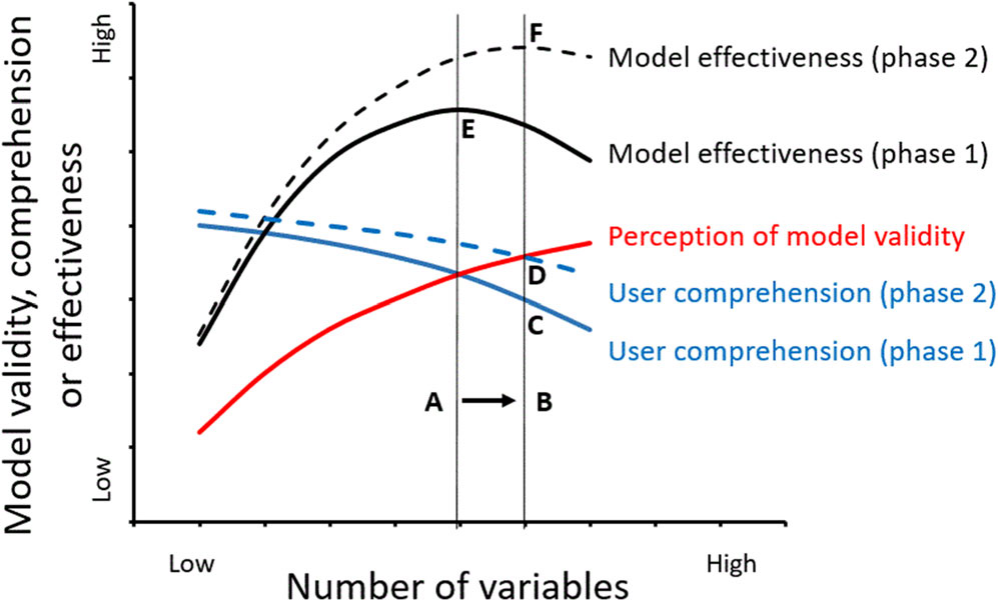
Schematic representation of the effect of the number of variables on the perceived model validity, user comprehension, and model effectiveness as a learning tool. In phase 2, the number of variables increased (A to B), and user comprehension was improved by documentation and a clearer interface (C to D), creating a new optimum for model effectiveness (E to F)

We observed in the workshops that an increase in the number of management variables tended to improve participants' perception of the validity of the model (the red line in Figure 5). For example, the lack of a yield response to *N* was seen as a limitation during the first phase of model development. However, the capacity of an individual user to comprehend the model processes and outputs can also decline as more variables are added (the blue line in Figure 5). For example, if the model is too complex the user may no longer understand how the model works (Brugnach et al., 2008) and reject the tool altogether (Kolkman et al., 2016). If we assume that the effectiveness of a model as a learning tool is the product of its validity and understanding, then there can be a number of variables at which the model reaches a point of maximum effectiveness (Point E in Figure 5).

Our experience in the second phase showed that by providing a clearer introduction, improved documentation and an improved layout of the ‘Control’ worksheet it was possible to both increase the model complexity (A to B in Figure 5) and maintain user understanding (C to D), creating a new optimum for model effectiveness (E to F). We observed that many participants were able to explore the theory and assumptions underpinning the grass model, leading to a deeper understanding (Hamilton et al., 2019). For example, in line with the theoretical analysis described by Parsons (1992), we used the model to establish that there is an optimal level of cutting interval to maximize green leaf yield (Figure 3a), whilst total biomass yield (including stem) approaches a plateau as cutting interval increases (Figure 3b).

Participants' ability to use the model was partly determined by existing abilities, e.g. capacity and familiarity with using spreadsheet tools, and willingness to use such tools (Smajgl, 2015). One of the benefits of a spreadsheet environment is that algorithms are potentially more accessible to the user than in some other software environments. Another way to improve the accessibility and transparency of the model is to provide full documentation of the rationale for the tool and the underlying science, the intended application domain and its limitations (Crout et al., 2008). In the second year, we provided a teaching guide (Burgess et al., 2020) with the LINGRA‐N‐Plus model, which allowed users to explore the theory and the relation between management decisions and the outputs from the ‘Calculations’ worksheet. We propose that this allowed us to shift the comprehension curve to the right (Figure 5). A final way to improve comprehension is to improve the user interface, as seen in the second round of workshops, where more users found LINGRA‐N‐Plus easier to use and understand than the initial model, with some identifying it as ‘excellent as a teaching or demonstration tool’ (Table 3). This objective of including sufficient detail in responses, whilst maintaining comprehension, interpretability and explanation for the user is also the focus of a growing area of research in the field of machine learning and artificial intelligence (AI), called ‘explainable AI’ (Doran et al., 2018).

### 4.4 Different perspectives of users

The workshops showed that students and practitioners used the tool differently, with the former often focusing on the mechanistic understanding of processes by asking ‘how?’ and ‘why?’ and the latter focusing more on the instrumental value of the tool, by asking ‘how much?’ and ‘when?’. This reflects their different perspectives and objectives. Students, for example, want to learn the key principles and mechanisms. Practitioners, who already have this understanding and experiential knowledge are looking for more quantified and predictive information for specific circumstances.

These perspectives translated into preferences for tool format. Students tended to place greater emphasis on the interactive nature of the tool which revealed causal relationships and mechanisms, while consultants were more interested in a simpler version or simplified interface of the tool for quick results. They also placed a greater emphasis on the validation of the model which for them is central to the relevance of the outputs.

Gilbert et al. (2018) noted that the appropriate balance between the model's complexity and transparency depends on the user's capacity and expectations for its use. In a teaching environment, it may be important that the model is not a black box and that users can access and trace the logic of its inner workings (Matthews et al., 2011; Stirling, 2010). By contrast, in a more applied situation, the ease of inputting site specific data and retrieving solely yield and quality outputs may be important. Hence, some users were interested in developing the tool as a phone‐based app (Table 3).

### 4.5 Effectiveness of the pedagogy

In relation to the different perspectives, students and practitioners learned in different ways. How can we use classroom teaching to enhance students' ability to implement the theory learned about grass growth and the role played by the LINGRA grass models? To help students become capable and competent future practitioners/consultants, they need to acquire knowledge and comprehension and develop skills (Wrenn & Wrenn, 2009).

During our first year of workshops, the students found that LINGRA can be used to describe the effect of different harvest intervals on green leaf yield. It was shown for instance, that under optimal *N* and water conditions, harvesting every 22 days would maximize green leaf yield. By contrast, in the second year when we used the improved model (LINGRA‐N‐Plus), we were able to show that the harvest interval that maximized green leaf yields was different from the harvest interval to maximized total harvestable dry matter (Figure 3b).

### 4.6 Benefits of stakeholder engagement including enhanced learning

A common failure of some of the early models and tools was that they were developed by researchers alone and did not take sufficient account of the perspectives of users and other stakeholders (Cox, 1996). The LINGRA‐N‐Plus model was tested and evaluated with students and practitioners at an early stage, and their input helped to optimize the tool's potential. The use of active stakeholder engagement in model development is increasingly common and can improve the credibility, relevance and usability of the model. It can also enhance collective learning. Participatory development of tools has proven to be an effective way to achieve learning with advisors and farmers and to include their own knowledge (Jakeman et al., 2006; McCown et al., 2012; Lundström & Lindblom, 2018). In our study, practitioners appeared to be energized by the collective learning from workshop discussions and appreciated the chance to learn about outcomes from different management options and scenarios, and to exchange ideas prompted by the tool. The value of simulation and exploring ‘What ifs’ with farmers, and the potential of building capacity in the advisory community through tool development and use is well known (Eastwood et al., 2012; Martin et al., 2011).

Stakeholders can be engaged in the form of knowledge provision, model selection and development, data collection and integration, scenario development, interpretation of results and development of policy alternatives. It is generally recognized that engaging participants in as many of these phases as possible and as early as possible, improves the value of the resulting model in terms of its usefulness to decision makers, its educational potential for the public and its credibility within the community (Beirele & Cayford, 2002; Korfmacher, 2001; Reed, 2008). Engaging with stakeholders is likely to increase the chances that model outputs, and their strengths and weaknesses will be understood at a deep rather than superficial level (Voinov & Bousquet, 2010). Through this engagement, the required level of model complexity, accuracy and scope can emerge from deliberative processes (Bellocchi et al., 2015; Colvin et al., 2014). In this respect, social scientists who are familiar with both the research and stakeholder communities can act as ‘bridges’ between different groups (Sterk et al., 2011). As Kipling et al. (2016b) pointed out, the challenge for modellers is to follow the above process to create models that are both ‘user friendly’ and robust at appropriate levels of complexity. If non‐scientists cannot parameterize, understand, or use the model, it will not be applied by local decision makers to solve real problems (Hamilton et al., 2019; Voinov & Bousquet, 2010).

### 4.7 Limitations and future developments of the tool

Since the above study, we have been able to demonstrate that LINGRA‐N‐Plus is able to predict annual grass yields under a range of pedo‐climatic and management conditions across England and Wales (Giannitsopoulos et al., 2021). This showed that LINGRA‐N‐Plus provided improved grass yields predictions (Giannitsopoulos et al., 2021) compared to the original LINGRA‐N (Wolf 2012), and similar predictions compared to the Rothamsted LINGRA‐based grass model (Qi et al., 2017).

As with any modelling process, there are still ways to improve the model. For instance, frameworks currently exist to interpret model performance and uncertainties and to simulate C fluxes in cropping and grassland systems at a variety of distant and contrasting sites (Sándor et al., 2020). Also, the model does not currently account for *N* leaching (or any other *N* transformations or losses) and assumes that only 70% of the applied *N* is available. The model has only been validated for perennial ryegrass production and lacks estimates of forage quality or predictions of multi‐species response. In fact, a recent study (van Oijen et al., 2020), highlighted the role of plant diversity in regulating the processes underlying the ecosystems services provided by multi‐species grasslands. Hence, based on the feedback from the participants, future developments of LINGRA‐N‐Plus could include calibration for different grass species, a routine to describe forage quality and the capacity to simulate more detailed *N* and *C* dynamics.

Recent research has also underlined that combining unmanned aerial systems (UAS) with multispectral cameras can allow for an optimal observation system capable of deploying machine learning algorithms for near real‐time mapping of perennial ryegrass dry matter (Togeirode Alckmin et al., 2022). As such technological solutions and efforts progress, they will have the potential to provide more data in an accurate and automated way with regards to, for instance, grass biomass assessments. This may be important for pasture monitoring or e.g., when grass yield data are needed to calibrate and validate different grass growth simulation models.

## 5 CONCLUSIONS

Computer‐based learning and decision‐making tools can improve the effectiveness of university teaching and consultant training by engaging students and practitioners. The well‐established simulation tool LINGRA‐N was implemented in a spreadsheet environment and expanded (LINGRA‐N‐Plus) to account for its application to different *N* levels and harvest intervals. A series of workshops showed that such a tool was useful in stimulating discussions and improving understanding of the theory and practice of grassland management. The major strengths of the resulting tool were its benefits as an effective teaching tool that could also prompt practitioner learning, the ease of use and understanding, the immediate visualization of results, and the efficient access to inputs, calculations and outputs. Some tended to find model complexity confusing, whilst others wished to increase functionality. Some aspects can be made more accessible through the intuitive design of a user‐friendly interface. The tool and associated workshops, both of which can be developed further, are proposed as an innovative way to explore different grassland management interventions in higher education and professional learning.

## CONFLICT OF INTEREST

The authors declare that they have no conflict of interest.

## Data Availability

The model described in the paper and the data associated with the paper can be accessed through Cranfield Online Research Data (CORD) repository system using the following link: https://doi.org/10.17862/cranfield.rd.11359613
